# Autism is associated with reduced ability to interpret grasping actions of others

**DOI:** 10.1038/s41598-017-12995-z

**Published:** 2017-10-04

**Authors:** Marco Turi, Filippo Muratori, Francesca Tinelli, Maria Concetta Morrone, David C. Burr

**Affiliations:** 1 Department of Translational Research on New Technologies in Medicine and Surgery, University of Pisa, Pisa, Italy; 2 Fondazione Stella Maris Mediterraneo, Chiaromonte, Potenza, Italy; 3 IRCCS Stella Maris Foundation, Pisa, Italy; 4 Department of Clinical and Experimental Medicine, University of Pisa, Pisa, Italy; 5 Department of Developmental Neuroscience, Stella Maris Scientific Institute, Pisa, Italy; 6 Robotics, Brain & Cognitive Sciences Department, Italian Institute of Technology, via Morego 30, 16163 Genoa, Italy; 7 Department of Neuroscience, Psychology, Pharmacology and Child Health, University of Florence, Florence, Italy; 8 CNR Neuroscience Institute, Pisa, Italy

## Abstract

We investigated the ability of children with ASD to discriminate a small cylinder from a large cube by observing a point-light movie of an actor grasping the object, either from an allocentric or egocentric viewpoint (observing action of others or self). Compared with typically developing controls, high functioning autistic children showed a strong selective impairment in this task, but only with the allocentric viewpoint, where thresholds were twice as high: egocentric thresholds were similar to age- and ability-matched controls. The magnitude of the impairment correlated strongly with the degree of symptomology (R^2^ = 0.5). The results suggest that children with ASD might be impaired in their ability to predict and infer the consequences of others’ movements, which could be related to the social-communicative deficits often reported in autism.

## Introduction

Autism is a neurodevelopmental disorder characterised by difficulties in social interaction and communication, as well as a restricted repertoire of interests and repetitive stereotyped behaviours. The condition is also associated with a range of non-social features, including both *hyper*sensitivity and *hypo*sensitivity to perceptual stimuli, and sensory seeking behaviours such as attraction to light, intense looking at objects and fascination with brightly coloured objects. These sensory atypicalities, which now form part of the diagnostic criteria for autism^1^, can have debilitating effects on the lives of autistic people and their families^2,3^. Movement atypicalities have been also linked with autism as far back as the work of Kanner^4^, who noted motor abnormalities such as ‘sluggish’ reflexes, ‘clumsy’ gait and an absence from an early age of anticipatory postures when being picked up^5^. Both gross and fine motor deficits are prevalent in ASD^6^, and include impairments in basic motor control^7^, abnormal patterns of motor learning^8^ and disturbance in the reach-to-grasp- movement^9^. There is also evidence for impairment of estimation of action capability, or *affordance* ^10^. This study investigates further the processes underlying affordance of autistic children, by investigating their ability to judge the size of objects from observing action from different perspectives.

The ability to perceive and interpret the actions of others is crucial for survival in a social environment. Human newborns show a selective preference for motion patterns generated by other living organisms, termed biological motion^11^. Some studies have suggested that this basic ability may be impaired in children with ASD^12,13^, which could contribute to the cognitive and social consequences of autism in later life^14^. However, not all studies have reported lower sensitivity in detecting biological motion in ASD. For example Murphy, Brady, Fitzgerald, & Troje^15^ and Saygin, Cook, & Blakemore^16^ found no difference between thresholds for biological motion for autistic and typically developing children. More recently Cusack, Williams, & Neri^17^ showed that differences between autistic observers and controls disappeared when they normalized thresholds for biological motion. By dividing biological motion discrimination thresholds with those for discrimination of inverted walkers it factor out any generalized attentional deficits or limitations associated with executive function during different perceptual tasks, which may be the limiting factor in autism.

It has long been known that perceptual and motor systems are tightly linked: action influences perception and perception influences action^18^. Many studies have pointed to a strong interconnection between motor and visual information, showing that the motor system can influence basic visual processing to improve perceptual skills^19–23^. Even the programming of a simple action can modulate visual thresholds^24^, and introducing a motor load has been shown to modulate perceptual judgments about the weight of an object being lifted by an actor^25^ or the speed of a walker^26^.

Traditional models of action-understanding emphasize that long-term experience in seeing a wide array of actions allows for effective anticipation or prediction of action^27^. Knoblich and colleagues^28,29^ have suggested that, during observation of action, the motor system activates in the observer action codes associated with the observed motor commands. The closer the match between the observer’s motor repertoire and the observed action, the better will be the understanding and anticipation of the sensory consequences of the unfolding action^28^.

The influence of the motor system on perception can also be revealed by studying the effect of viewpoint on perceiving biological motion^30^. Campanella and colleagues^31^ recently investigated the ability of young adults to discriminate object size by observing a point-light movie of an actor grasping an object, either from an allocentric (consistent with observing the action of others) or egocentric (consistent with observing the action of oneself) viewpoint. They showed that the discrimination was better when the action was observed from an egocentric viewpoint. In addition, when the subjects observed their own previously filmed actions the performance was even better. In any of those conditions, discriminate of object shapes were possible. Several additional controls demonstrated that the effect was not driven by spatial cues, such as the distance of the fingers at contact time or the maximum grip aperture or the grasping trajectory that could be performed from above or from the side of the object. The study was also extended to a large sample of typically developing children, ranging from 5 to18 years, to monitor developmental trajectory^32^. Children under 7 years of age failed to discriminate object size by observation of action, from either egocentric or allocentric viewpoints. The ability improved progressively up till about 18 years, with an advantage for the egocentric viewpoint emerging after about 9 years (as reported for adults^31^).

It has been suggested that impairments in understanding and imitation of action in individuals with ASD can be explained by an abnormal mirror neuron system (MNS)^33–35^, more neutrally termed the action observation network (AON)^36^. This network contains neurons that fire during action execution as well as observation of others performing the same actions^37,38^. It is thought to generate a simulation circuit that allows the association between one’s own actions with the action of others, and hence could play an important role in understanding action and in imitation, social interaction, and language comprehension^39–41^. Using electromyographic (EMG) recordings, Cattaneo *et al*.^42^ demonstrated that autistic children show reduced abilities in predicting the consequences both of their own actions, and those of others. This and several other studies^33,43^ suggest that MNS may be impaired in autism. However, while the notion that the MNS may be impaired in autism currently enjoys a good deal of popularity, it is important to emphasise that there is also considerable evidence against mirror-neuron dysfunction in ASD^44–49^.

During social interaction, prediction of the actions of others is important to adjust our movements to give appropriate and coordinated responses. The inability to automatically integrate social information and to use it to predict the actions of others could be due to a deficit in predictive coding. It has been recently suggested that the unique perceptual experience of individuals with autism may be accounted for within a Bayesian computational model of perceptual inference, proposing that they could make reduced use of *priors* or predictive information^50^. The Bayesian class of theories – including predictive coding and other generative models^51–53^ – assumes that perception is an optimized combination of sensory data (the *likelihood*) and top-down influences based on previous perceptual history (the *prior*). This process may be atypical in autism, in that the *priors* may be under-weighted, or less utilized than in typical individuals. This theory has been reinforced by several others along similar lines^54–58^, and has received empirical support from studies showing diminished adaptation in autistic individuals in the processing of face^59,60^ and non-face stimuli^61–65^.

Here we aimed to understand whether the perception of the goal of the action of others was specifically impaired in autistic children. We used the same biological motion stimuli of Tinelli *et al*.^32^ where reaching action towards invisible large or small object were displayed in allocentric or egocentric perspective. We find that autistics do show a selective impairment in estimating size from the allocentric perspective, consistent with a specific deficit in the understanding of the action of others by visual observation.

## Results

**Figure 1:**
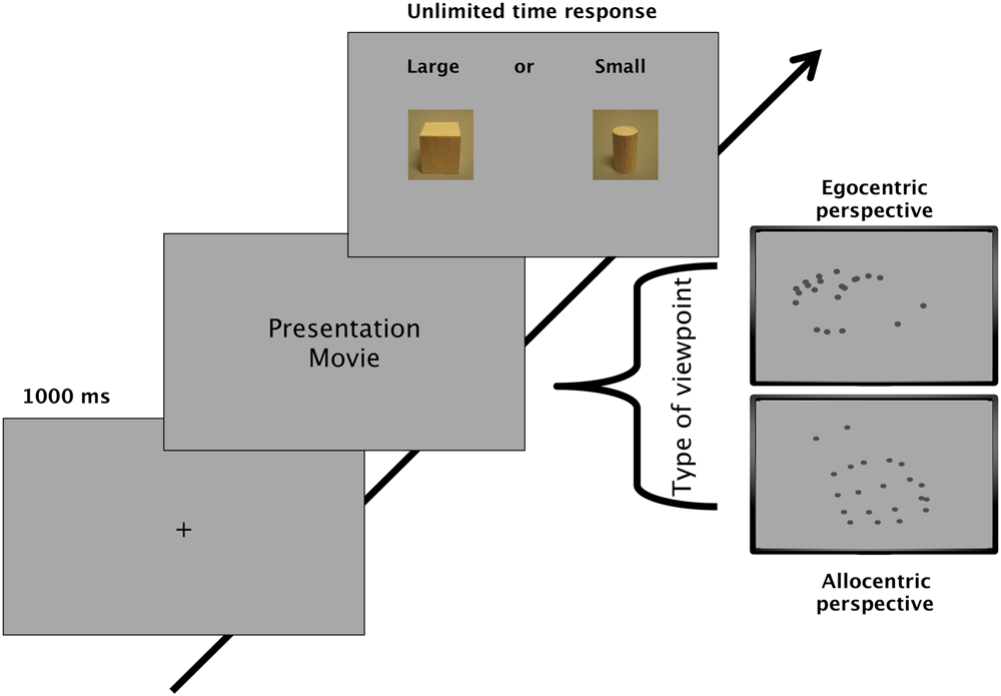
Examples of stimuli and procedures used in the experiments. Object identification task: biological motion movies representing a hand grasping non-visible objects of different size and shape displayed either from an egocentric (observing self-action, top example) or from an allocentric point of view (observing others’ action, bottom example). Subjects were asked to indicate whether the goal of the reach-and-grasp movement was towards a small cylinder or large cube.

**Figure 2:**
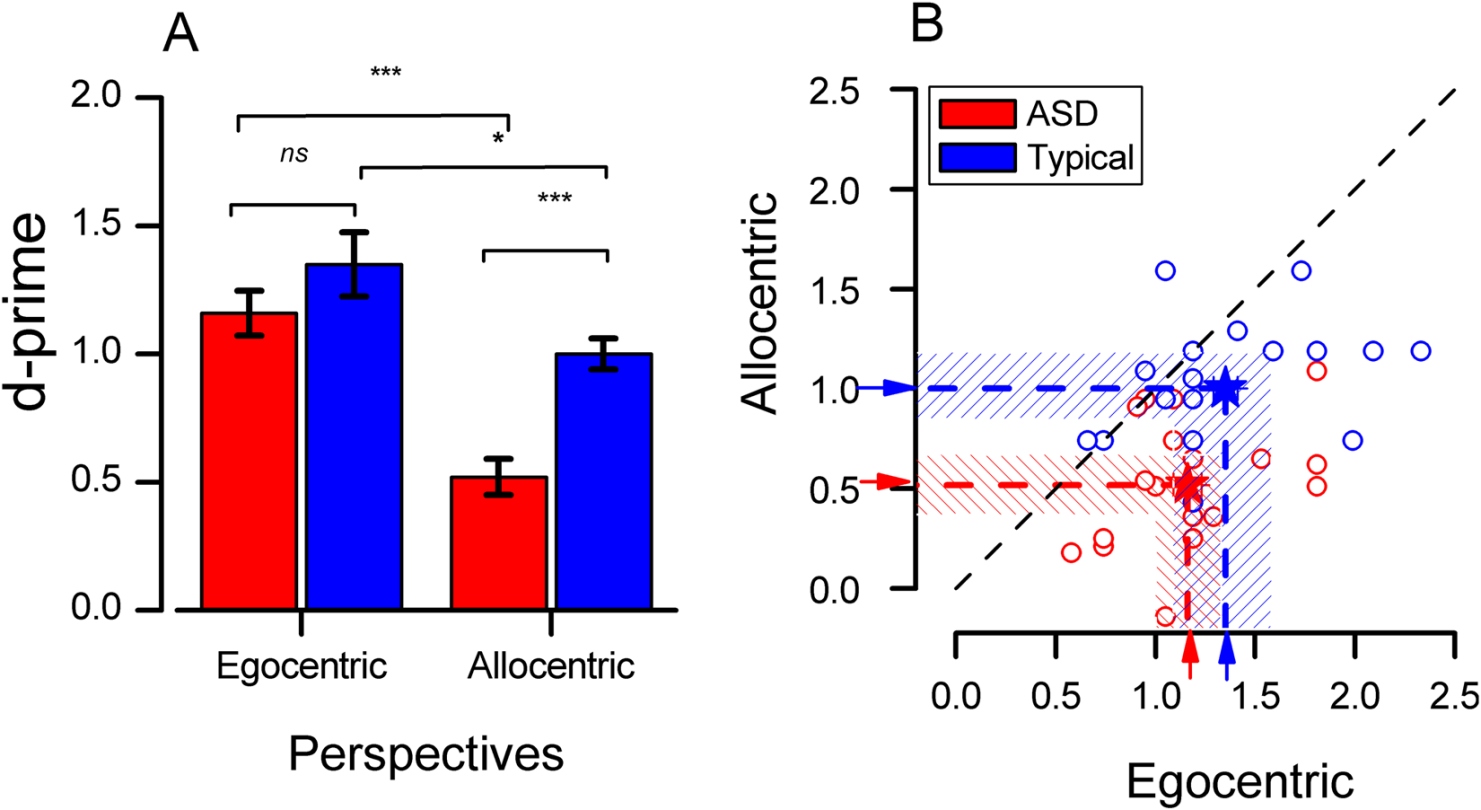
Group differences in discrimination performance. (**A**) Mean sensitivity (d’) in the egocentric and allocentric conditions for the two groups. Error bars correspond to ± 1 SEM. Stars show significance of t-tests: ***p < 0.001, **p < 0.01, *p < 0.05 and ns non-significant. (**B**) Scatterplot of sensitivity (measured as d’) in the egocentric perspective plotted against sensitivity in the allocentric perspective for all participants (children with ASD, red symbols; typical children, blue symbols). The color-coded arrows indicate the mean of the two groups and shaded areas 95% confidence intervals. Accuracy was similar between typically developing comparison children and children with ASD when the stimulus was displayed in egocentric view, but much lower for the ASD group in the allocentric view.

Subjects observed biological motion movies of a hand grasping an invisible object, and guessed whether the goal of the movement was towards a small cylinder or large cube (see methods and Fig. 1). We calculated discrimination performance for each participant, expressed as discrimination index *d’*, for observing the action from an egocentric or allocentric viewpoint, pooling both grasping directions (sideways or from above). Figure 2 shows mean *d’* for the egocentric and allocentric conditions for both ASD and typically-developing (TD) children. A mixed-design ANOVA analysis with view (egocentric, allocentric) as a repeated-measures factor and group (autism and typical) as a between-participants factor on the discrimination performance yielded significant main effects of view (F(1, 35) = 45.01, p < 0.0001), with better performance for stimuli displayed in the egocentric view (M = 1.25 SEM = 0.07) than the allocentric view (M = 0.78 SEM = 0.05). There was also a significant effect of group (F(1,35) = 11.46, p = 0.002), with ASD children having lower sensitivity (M = 0.84 SEM = 0.07) than TD children (M = 1.18 SEM = 0.07). These main effects were qualified by a significant interaction between view and group (F(1,34) = 5.59, p = 0.024). Bonferroni-corrected post hoc t-test (shown by the stars of Fig. 2A) showed that children with ASD had a significantly lower sensitivity than TD children in allocentric view (ASD: M = 0.52, SD = 0.31; TD: M = 1.03, SD = 0.30: t_(35)_ = −4.96 p < 0.0001), but there was no difference in the egocentric condition (ASD: M = 1.16, SD = 0.35; TD: M = 1.35, SD = 0.49; t_(35)_ = −1.28 p = 0.22). Both groups showed a higher sensitivity in the egocentric view compared with the allocentric view (ASD: t_(18)_ = 7.25, p < 0.0001; TD: t_(17)_ = 2.75 p = 0.01).

We further investigated the effect of grasping direction (grasping from the side or from above) on object identification with a mixed-design ANOVA on *d’*, with group as the between-participants factor and grasping configuration (Side and Above) as the within-participants factor. We found that there was a significant main effect of group (F(1, 35) = 7.21, p = 0.01), with a better general discrimination of the size in the typical group (TD: M = 1.08 S.E.M. = 0.07; ASD: M = 0.79 S.E.M. = 0.07). The ANOVA revealed no statistically significant main effect of grasping direction (F(1, 35) = 0.53, p = 0.47) nor a significant interaction (F(1, 35) = 0.26, p = 0.61).

**Figure 3:**
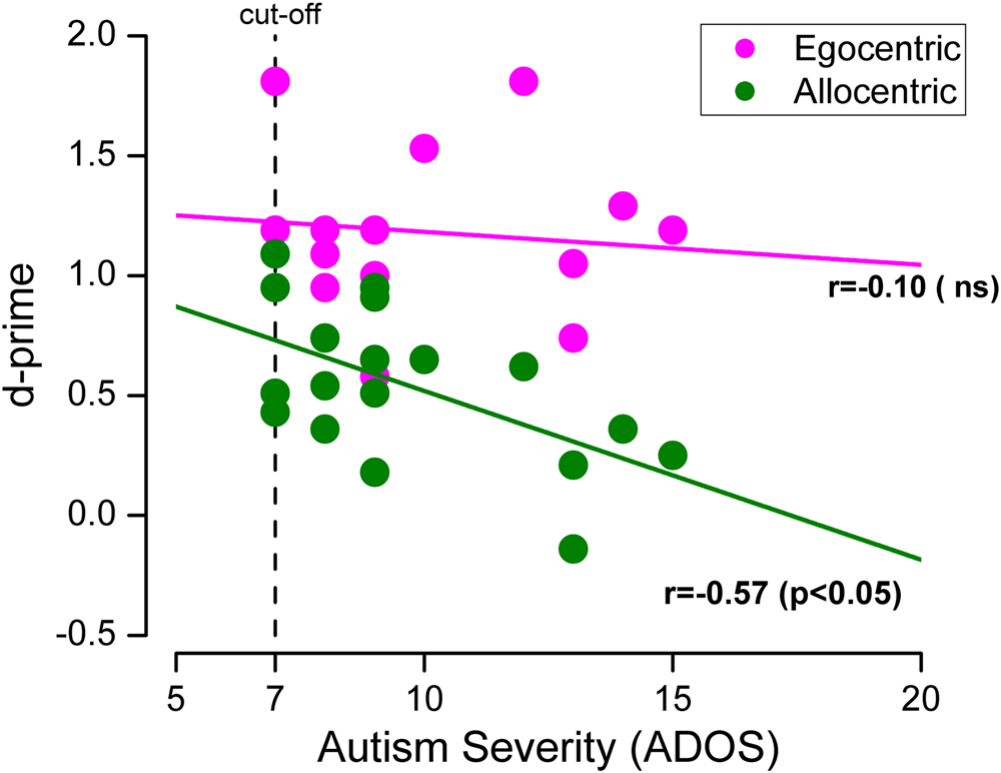
Relationship between discrimination and autism severity. Discrimination sensitivity (d’) as function of autism severity (ADOS social-communication total score) for all autistic children (Egocentric view: purple; Allocentric view: green). The colour-coded lines show the correlation within each perspective.

We then examined the relationship between sensitivity and measures of autism symptomatology (ADOS social-communication total score). Figure 3 shows that sensitivities in the allocentric condition correlated negatively with the severity of symptoms (r = −0.57, p = 0.013, Log Bayes Factor = 0.63). However, there was no correlation for the egocentric condition (r = −0.10, p = 0.69, Log Bayes Factor = −0.74). Log Bayes Factors greater than 0.5 are considered reliable evidence in favour of the hypothesis (correlation with allocentric sensitivity), and factors less than −0.5 evidence for the null hypothesis (no correlation). We found no significant correlation between sensitivity and verbal and non-verbal abilities in either group (all p > 0.05, Log Bayes Factors ranging from −0.13 to −0.75).

## Discussion

The aim of the present study was to investigate the ability of autistic children to discriminate objects by observing a point-light movie of an actor grasping the object, either from an allocentric or egocentric viewpoint (observing the action of others or self). Children with ASD performed similarly to typically developing children when viewing from the egocentric perspective. However, allocentric judgements were selectively impaired with autism, and that only these correlated with the individual differences in autism symptoms. That sensitivity in the egocentric perspective is unimpaired demonstrates that autistic people do not have difficulties with interpreting biological motion per se, but do so when observing the actions of others. The ADOS assessment measures symptoms such as unusual eye-contact, poor social response and limited reciprocal interactions, which are all consistent with autistic individuals having difficulties in understanding the action of others.

Although not all researchers agree^44–49^, much behavioural and imaging research has provided substantial support for mirror-neuron dysfunction in ASD during action observation and motor control^33–35,42,66–70^. Recently Kilner and colleagues^71^ have proposed a predictive coding account for the mirror neuron system, suggesting that during action observation an internal model is generated with a prior prediction of the intention of the person whom we are observing, and that this representation is transmitted to the superior temporal sulcus (STS) and parietal brain areas^72^. The deficit in inferring the size of an object from allocentric observation is congruent with a deficit in the mirror neuron system, which in turn is consistent with the more general concept of a deficit in prediction. This agrees with the ideas that autistic perception can be understood in terms of reduced use of Bayesian priors^50^, and in prediction of action^57^.

Another possible explanation for the results could be that visual system experience plays an important role. Infants spend a large proportion of their time during early development watching their own limb movements^73–75^, as well as those of others. There is evidence that infants with autism may spend longer watching their own movements, compared with those of others, which could lead to the difference in sensitivity between perceiving allocentric and egocentric viewpoints. There is also evidence that the basic movements of autistic adults are different from typically developing individuals, showing higher acceleration and jerk, and that these differences are correlated with biases in perceiving biological motion^23^. However, this should have caused poorer sensitivity for both viewpoints, rather than a selective impairment for the allocentric view as we observed here.

Whatever the detailed explanation, the results of this study show that children with autism have reduced ability to judge size of objects from observing the actions of others, especially when viewed from an allocentric viewpoint. This is consistent with the idea that children with ASD may have impaired capacity to predict and infer the consequence of movements of others. It is also consistent with much evidence showing that movement kinematics are significantly altered in autism^76^, which could lead to an atypical movement representation, which is likely to impact on the perception, prediction and understanding the action of others. Importantly, the misunderstanding of actions is reciprocal, as neurotypical observers also fail to understand well the movements of autistic individuals^77^. All this may contribute to the impaired social social-communication often reported in autism.

## Material and Methods

### Participants

We tested 19 children with autistic spectrum disorder (ASD) aged 8–17 years (mean 11.5 years, SD 2.2) and 18 typically developing children (mean 11.9 years, SD 2.7). All children with autism met Diagnostic and Statistical Manual of Mental Disorders, Fourth Edition (DSM-IV) criteria^1^ for autism, according to an independent clinician, and met criteria for an ASD on the Autism Diagnostic Observation Schedule – 2^nd^ edition (ADOS-2;^78^), see Table 1. The ADOS were administered by clinically qualified neuro-psychologists, two of which had also a research-level qualification, employed by the child neuropsychiatry department of the hospital. The groups were matched for chronological age, t_(35)_ = −0.51, p = 0.61, and full-scale IQ, t_(35)_ = −0.36, p = 0.71 (independent samples t-test, two tailed), as measured by the Wechsler Abbreviated Scales of Intelligence (WASI^79^), see Table 1 for details. All children had a total IQ score above 80 and were thus considered “cognitively able”. No child had a medical or developmental disorder other than ASD, as reported by parents, nor was on medication. Also, no typically developing child had with a current or past medical or psychiatric diagnosis, as reported by parents. All children had normal visual acuity. Participants were tested individually in a quiet room either at home or at the Stella Maris Scientific Institute. They and their parents gave informed consent to participate in the study, which was approved by the regional paediatrics ethics committee (*Comitato Etico Pediatrico Regionale—Azienda Ospedaliero-Universitaria Meyer—Firenze*) and are in line with the declaration of Helsinki.

**Table 1:** Demographic Information.

	Children with ASD	Typical developing children	*t-test*
**Gender (male: female)**	16: 3 (19)	14: 4 (18)	
**Age (years)**			
Mean (SD)	11.49 (2.24)	11.94 (2.73)	t_(35)_ = −0.51, p = 0.61
Range	8–16.5	8–17	
**Full Scale IQ**			
Mean (SD)	105.9 (16.68)	107.6 (9.21)	t_(35)_ = −0.36, p = 0.71
Range	80–141	104–124	
**ADOS Score**			
Mean (SD	9.66 (2.55)	—	
Range	7–15	—	

Descriptive statistics for developmental variables for children with autism and typically developing children.

### Stimuli

The visual stimuli comprised point-light biological motion movies representing a hand grasping two different objects that were invisible to the observer (see Supplementary Information). The same movement was presented in the egocentric (observing self-action) and allocentric point of view (observing action of others), as shown in Fig. 1. The biological motion stimuli were those prepared previously^31^, where actors were recorded grasping two objects (a cube or a cylinder) with an array of cameras positioned to capture the action in the three-dimensional space, using 23 markers placed on the centre of the nails, joints of all digits, the dorsal aspect of the hand and the radial and ulnar styloid process. To change the perspective of the grasping movement, the three-dimensional motion was rotated around the azimuth by 180°, so the visual information presented in the allocentric and egocentric view is identical. The movie showed both a lateral view and a top view of the hand grasp of the objects. Each cube side was 6.5 cm, while the cylinder was 6.5 cm high and 4 cm wide. The motion was always presented in the centre of the screen, starting from either the bottom or the top of the display for the egocentric and allocentric perspectives, respectively.

We chose for our test two objects of different size (a large cube versus a small cylinder), and asked the children which was larger. Campanella *et al*.^31^ performed many controls to demonstrate that the information contained in the maximum grip aperture, peak velocity of finger aperture and percentage of time to maximal finger aperture were not used by the observer to discriminate objects of matched size: discrimination of the shape of two small or two large objects was never above chance. We used the same procedure used by Tinelli *et al*.^32^ to measure young children. To avoid confusion in the subject response, we associated the size with the shape of different objects: the subject had to discriminate between a large cube and a small cylinder. To ensure that the task was clear to the children, the operator first mimicked a reaching and grasping movement towards a real large cube and a small cylinder, stressing the difference in size of the two objects, then asked the subject to perform the same action. Only when it was clear that the children had understood the task did the operator proceeded with the collection of the trials. The biological motion movie of the schematic hand marked with black dots was displayed on a computer screen using the MATLAB Psychophysics toolbox^80^. The hand subtended about 13 × 15° of visual angle (for other details see the electronic supplementary materials of Campanella *et al*.^31^).

### General procedures

All visual stimuli were presented in a dimly lit room on a 15.4 inch Acer monitor with 1024 × 768 resolution at refresh rate of 60 Hz and mean luminance 60 cd/m^2^, viewed binocularly from 57 cm. Visual stimuli were displayed for 0.90 s ± 0.15 s. After the movies, a response page appeared and the subjects were required to respond by pointing to the object that was the goal of the reach-and-grasp movement. Each subject performed 5 training trials before data acquisition, then 50 trials per block, with condition order pseudo-randomized across subjects. No feedback was given, nor was there a time limit. Experimenters monitored gaze at all times to ensure subjects were fixating screen-centre. Half the trials were displayed with movement towards the small cylinder and half towards the large cube; half of each size was from the egocentric viewpoint, half allocentric, presented in randomized order. The number of trials with movies showing grasping from the side or above were nearly balanced (difference less than 10%), which is important to avoid that the discrimination could be based on the movement trajectory.

### Data analysis

Discrimination performance was measured as *d’*, defined as the difference between the z-scores of the hits and the false alarms which, for a two-alternative forced-choice design, corresponds to 1 for 76 per cent correct responses (threshold value) and of 0 to 50 per cent correct (chance level). To evaluate condition and group-specific differences in sensitivity, as well as their statistical interaction, we used a mixed repeated-measure ANOVA employing the within-subject factor ‘perspective’ (Egocentric versus Allocentric) and a between-subject independent variable ‘group’ (ASD versus typical). ADOS scores were measured for correlation analyses with sensitivity measurements to estimate the relationship between the severity of autistic symptoms and task performance. The relationships between perceptual as well as cognitive variables were measured by bivariate correlations.

### Data availability

The data that support the findings of this study are available from the corresponding authors upon request.

## Electronic supplementary material

Supplementary Information

Supplementary Videos S1

Supplementary Videos S2

## References

[1] American Psychiatric Association Arlington, V. A. P. P. Diagnostic and statistical manual of mental disorders (5th ed.) (2013).

[2] Bagby MS, Dickie VA, Baranek GT (2012). How Sensory Experiences of Children With and Without Autism Affect Family Occupations. Am J Occup Ther.

[3] Williams, D. *Somebody somewhere: breaking free from the world of autism*. 1st edn, (Times Book, 1994).

[4] Kanner L (1943). Autistic disturbances of affective contact. Nervous Child.

[5] Flanagan JE, Landa R, Bhat A, Bauman M (2012). Head lag in infants at risk for autism: a preliminary study. Am J Occup Ther.

[6] Fournier KA, Hass CJ, Naik SK, Lodha N, Cauraugh JH (2010). Motor coordination in autism spectrum disorders: a synthesis and meta-analysis. Journal of autism and developmental disorders.

[7] Jansiewicz EM (2006). Motor signs distinguish children with high functioning autism and Asperger’s syndrome from controls. Journal of autism and developmental disorders.

[8] Haswell CC, Izawa J, Dowell LR, Mostofsky SH, Shadmehr R (2009). Representation of internal models of action in the autistic brain. Nature neuroscience.

[9] Mari M, Castiello U, Marks D, Marraffa C, Prior M (2003). The reach-to-grasp movement in children with autism spectrum disorder. Philosophical transactions of the Royal Society of London. Series B, Biological sciences.

[10] Linkenauger SA, Lerner MD, Ramenzoni VC, Proffitt DR (2012). A perceptual-motor deficit predicts social and communicative impairments in individuals with autism spectrum disorders. Autism research: official journal of the International Society for Autism Research.

[11] Simion F, Regolin L, Bulf H (2008). A predisposition for biological motion in the newborn baby. Proceedings of the National Academy of Sciences of the United States of America.

[12] Blake R, Turner LM, Smoski MJ, Pozdol SL, Stone WL (2003). Visual recognition of biological motion is impaired in children with autism. Psychological science.

[13] Klin A, Lin DJ, Gorrindo P, Ramsay G, Jones W (2009). Two-year-olds with autism orient to non-social contingencies rather than biological motion. Nature.

[14] Kaiser MD, Shiffrar M (2009). The visual perception of motion by observers with autism spectrum disorders: A review and synthesis. Psychon B Rev.

[15] Murphy P, Brady N, Fitzgerald M, Troje NF (2009). No evidence for impaired perception of biological motion in adults with autistic spectrum disorders. Neuropsychologia.

[16] Saygin, A. P., Cook, J. & Blakemore, S. J. Unaffected Perceptual Thresholds for Biological and Non-Biological Form-from-Motion Perception in Autism Spectrum Conditions. *Plos One* **5**, doi:ARTN e1349110.1371/journal.pone.0013491 (2010). 10.1371/journal.pone.0013491 PMC2956672 20976151

[17] Cusack JP, Williams JHG, Neri P (2015). Action Perception Is Intact in Autism Spectrum Disorder. Journal of Neuroscience.

[18] Goodale M, Milner AD (1992). Separate pathways for perception and action. TINS.

[19] Aglioti SM, Cesari P, Romani M, Urgesi C (2008). Action anticipation and motor resonance in elite basketball players. Nature neuroscience.

[20] Calvo-Merino B, Grezes J, Glaser DE, Passingham RE, Haggard P (2006). Seeing or doing? Influence of visual and motor familiarity in action observation. Current biology: CB.

[21] Casile A, Giese MA (2006). Nonvisual motor training influences biological motion perception. Current biology: CB.

[22] Arrighi R, Cartocci G, Burr D (2011). Reduced perceptual sensitivity for biological motion in paraplegia patients. Current biology: CB.

[23] Cook JL, Blakemore SJ, Press C (2013). Atypical basic movement kinematics in autism spectrum conditions. Brain.

[24] Tomassini A, Spinelli D, Jacono M, Sandini G, Morrone MC (2015). Rhythmic oscillations of visual contrast sensitivity synchronized with action. The Journal of neuroscience: the official journal of the Society for Neuroscience.

[25] Hamilton A, Wolpert D, Frith U (2004). Your own action influences how you perceive another person’s action. Current Biology.

[26] Jacobs A, Shiffrar M (2005). Walking perception by walking observers. J Exp Psychol Human.

[27] Mulligan D, Hodges NJ (2014). Throwing in the dark: improved prediction of action outcomes following motor training without vision of the action. Psychological research.

[28] Knoblich G, Flach R (2001). Predicting the effects of actions: interactions of perception and action. Psychological science.

[29] Knoblich G, Seigerschmidt E, Flach R, Prinz W (2002). Authorship effects in the prediction of handwriting strokes: evidence for action simulation during action perception. The Quarterly journal of experimental psychology. A, Human experimental psychology.

[30] Bach P, Fenton-Adams W, Tipper SP (2014). Can’t touch this: the first-person perspective provides privileged access to predictions of sensory action outcomes. Journal of experimental psychology. Human perception and performance.

[31] Campanella F, Sandini G, Morrone MC (2011). Visual information gleaned by observing grasping movement in allocentric and egocentric perspectives. Proc Biol Sci.

[32] Tinelli F, Cioni G, Sandini G, Turi M, Morrone MC (2017). Visual information from observing grasping movement in allocentric and egocentric perspectives: development in typical children. Exp Brain Res.

[33] Iacoboni M, Dapretto M (2006). The mirror neuron system and the consequences of its dysfunction. Nature Reviews Neuroscience.

[34] Oberman LM, Ramachandran VS (2007). The simulating social mind: The role of the mirror neuron system and simulation in the social and communicative deficits of autism spectrum disorders. Psychol Bull.

[35] Williams JHG, Whiten A, Suddendorf T, Perrett DI (2001). Imitation, mirror neurons and autism. Neurosci Biobehav R.

[36] Van Overwalle F, Baetens K (2009). Understanding others’ actions and goals by mirror and mentalizing systems: a meta-analysis. NeuroImage.

[37] Gallese V, Fadiga L, Fogassi L, Rizzolatti G (1996). Action recognition in the premotor cortex. Brain.

[38] Rizzolatti G, Fadiga L, Gallese V, Fogassi L (1996). Premotor cortex and the recognition of motor actions. Brain research. Cognitive brain research.

[39] Fogassi L (2005). Parietal lobe: from action organization to intention understanding. Science.

[40] Iacoboni M (2009). Imitation, empathy, and mirror neurons. Annual review of psychology.

[41] Rizzolatti G, Sinigaglia C (2010). The functional role of the parieto-frontal mirror circuit: interpretations and misinterpretations. Nature reviews. Neuroscience.

[42] Cattaneo L (2007). Impairment of actions chains in autism and its possible role in intention understanding. Proceedings of the National Academy of Sciences of the United States of America.

[43] Oberman LM (2005). EEG evidence for mirror neuron dysfunction in autism spectrum disorders. Brain research. Cognitive brain research.

[44] Bird G, Leighton J, Press C, Heyes C (2007). Intact automatic imitation of human and robot actions in autism spectrum disorders. Proc Biol Sci.

[45] Dinstein I (2010). Normal movement selectivity in autism. Neuron.

[46] Gowen E, Stanley J, Miall RC (2008). Movement interference in autism-spectrum disorder. Neuropsychologia.

[47] Hamilton AF, Brindley RM, Frith U (2007). Imitation and action understanding in autistic spectrum disorders: how valid is the hypothesis of a deficit in the mirror neuron system?. Neuropsychologia.

[48] Leighton J, Bird G, Charman T, Heyes C (2008). Weak imitative performance is not due to a functional ‘mirroring’ deficit in adults with Autism Spectrum Disorders. Neuropsychologia.

[49] Spengler S, Bird G, Brass M (2010). Hyperimitation of actions is related to reduced understanding of others’ minds in autism spectrum conditions. Biological psychiatry.

[50] Pellicano E, Burr D (2012). When the world becomes ‘too real’: a Bayesian explanation of autistic perception. Trends in cognitive sciences.

[51] Kersten D, Mamassian P, Yuille A (2004). Object perception as Bayesian inference. Annual review of psychology.

[52] Knill DC, Pouget A (2004). The Bayesian brain: the role of uncertainty in neural coding and computation. Trends in neurosciences.

[53] Mamassian, P., Landy, M. & Maloney, L. In *Probabilistic Models of the Brain: Perception and Neural Function* (eds R. Rao, B. Olshausen, & M. Lewicki) (Bradford Books, 2002).

[54] Friston KJ, Lawson R, Frith CD (2013). On hyperpriors and hypopriors: comment on Pellicano and Burr. Trends in cognitive sciences.

[55] Lawson, R. P., Rees, G. & Friston, K. J. An aberrant precision account of autism. *Frontiers in Human Neuroscience* **8**, 10.3389/fnhum.2014.00302 (2014). 10.3389/fnhum.2014.00302 PMC4030191 24860482

[56] Van de Cruys S, de-Wit L, Evers K, Boets B, Wagemans J (2013). Weak priors versus overfitting of predictions in autism: Reply to Pellicano and Burr (TICS, 2012). i-Perception.

[57] Sinha P (2014). Autism as a disorder of prediction. Proceedings of the National Academy of Sciences.

[58] Rosenberg A, Patterson JS, Angelaki DE (2015). A computational perspective on autism. Proceedings of the National Academy of Sciences.

[59] Pellicano E, Jeffery L, Burr D, Rhodes G (2007). Abnormal adaptive face-coding mechanisms in children with autism spectrum disorder. Current biology: CB.

[60] Pellicano E, Rhodes G, Calder AJ (2013). Reduced gaze aftereffects are related to difficulties categorising gaze direction in children with autism. Neuropsychologia.

[61] Turi M (2015). Children with autism spectrum disorder show reduced adaptation to number. Proceedings of the National Academy of Sciences of the United States of America.

[62] Turi, M., Karaminis, T., Pellicano, E. & Burr, D. No rapid audiovisual recalibration in adults on the autism spectrum. *Sci Rep-Uk* 6, Artn 2175610.1038/Srep21756 (2016). 10.1038/srep21756 PMC4761981 26899367

[63] van Boxtel JJ, Dapretto M, Lu H (2016). Intact recognition, but attenuated adaptation, for biological motion in youth with autism spectrum disorder. Autism research: official journal of the International Society for Autism Research.

[64] Lawson, R. P., Aylward, J., Roiser, J. P. & Rees, G. Adaptation of social and non-social cues to direction in adults with autism spectrum disorder and neurotypical adults with autistic traits. Developmental cognitive neuroscience, doi:10.1016/j.dcn.2017.05.001 (2017). 10.1016/j.dcn.2017.05.001 PMC6053619 28602448

[65] Lawson, R. P., Aylward, J., White, S. & Rees, G. A striking reduction of simple loudness adaptation in autism. *Sci Rep* **5**, 16157, doi:10.1038/srep16157 (2015). 10.1038/srep16157 PMC4633623 26537694

[66] Fabbri-Destro M, Cattaneo L, Boria S, Rizzolatti G (2009). Planning actions in autism. Exp Brain Res.

[67] Avikainen S, Wohlschlager A, Liuhanen S, Hanninen R, Hari R (2003). Impaired mirror-image imitation in Asperger and high-functioning autistic subjects. Current biology: CB.

[68] Dapretto M (2006). Understanding emotions in others: mirror neuron dysfunction in children with autism spectrum disorders. Nature neuroscience.

[69] McIntosh DN, Reichmann-Decker A, Winkielman P, Wilbarger JL (2006). When the social mirror breaks: deficits in automatic, but not voluntary, mimicry of emotional facial expressions in autism. Dev Sci.

[70] Rogers SJ, Hepburn SL, Stackhouse T, Wehner E (2003). Imitation performance in toddlers with autism and those with other developmental disorders. Journal of child psychology and psychiatry, and allied disciplines.

[71] Kilner JM, Friston KJ, Frith CD (2007). Predictive coding: an account of the mirror neuron system. Cognitive processing.

[72] Kilner JM, Friston KJ, Frith CD (2007). The mirror-neuron system: a Bayesian perspective. Neuroreport.

[73] Rochat P (1998). Self-perception and action in infancy. Exp Brain Res.

[74] van der Meer AL, van der Weel FR, Lee DN (1995). The functional significance of arm movements in neonates. Science.

[75] White BL, Castle P, Held R (1964). Observations on the Development of Visually-Directed Reaching. Child development.

[76] Cook, J. From movement kinematics to social cognition: the case of autism. *Philosophical transactions of the Royal Society of London. Series B, Biological sciences* **371**, doi:10.1098/rstb.2015.0372 (2016). 10.1098/rstb.2015.0372 PMC4843610 27069049

[77] Brewer R (2016). Can Neurotypical Individuals Read Autistic Facial Expressions? Atypical Production of Emotional Facial Expressions in Autism Spectrum Disorders. Autism research: official journal of the International Society for Autism Research.

[78] Lord, C. *et al*. *Autism Diagnostic Observation Schedule, Second Edition* (*ADOS-2*). (Western Psychological Services, 2012).

[79] Wechsler, D. *Wechsler Abbreviated Scale of Intelligence*. (The Psychological Corporation: Harcourt Brace & Company, 1999).

[80] Brainard DH (1997). The Psychophysics Toolbox. Spat Vis.

