# Supplementary material for: Autism is associated with reduced ability to interpret grasping actions of others
Source: Sci Rep. 2017 Oct 4;7:12687. doi: 10.1038/s41598-017-12995-z (PMC5627240; doi:10.1038/s41598-017-12995-z)
Supplement: Supplementary file 1 — Supplementary Information [file 41598_2017_12995_MOESM1_ESM.doc]

Online Supplementary Information

**Autism is associated with reduced ability to interpret grasping actions of others**

Marco Turi, Filippo Muratori, Francesca Tinelli, Maria Concetta Morrone, David C. Burr

Supplementary Videos:

Supplementary Videos S1 . **Example of the grasping biological movie from egocentric perspective**

Supplementary Videos S2 . **Example of the grasping biological movie from allocentric perspective**
